# Artificial Intelligence‐Based Approaches for Autism Screening Using Facial Images and DSM‐5 Checklist Data: A Methodological Study

**DOI:** 10.1002/hsr2.73360

**Published:** 2026-10-08

**Authors:** Fatemeh Mohammadi, Peyman Rezaei‐Hachesu, Afsoon Asadzadeh, Hassan Shahrokhi

**Affiliations:** ^1^ Department of Health Information Technology, School of Management and Medical Informatics Tabriz University of Medical Sciences Tabriz Iran; ^2^ Emergency and Trauma Care Research Center Tabriz University of Medical Sciences Tabriz Iran; ^3^ Autism and Related Neurodevelopmental Disorders Research Team Tabriz University of Medical Sciences Tabriz Iran; ^4^ Department of Psychiatry Tabriz University of Medical Sciences Tabriz Iran

**Keywords:** autism spectrum disorder, deep learning, DSM‐5, facial image analysis, screening

## Abstract

**Background and Aims:**

Artificial intelligence methods may support research on autism spectrum disorder (ASD)‐related screening, but they cannot replace comprehensive clinical assessment. We evaluated two image‐training scenarios public Kaggle data alone and a manually prepared combined training set containing Kaggle images plus local images and separately examined a structured DSM‐5 checklist model. A child‐level reanalysis was used to evaluate public‐to‐local transfer and local adaptation while ensuring that images from the same child were not assigned to different partitions.

**Methods:**

This methodological study used 268 retrospectively abstracted DSM‐5 records (134 ASD and 134 non‐ASD) and 162 local facial images from 92 children (43 ASD and 49 non‐ASD). The image experiment evaluated EfficientNet‐B2 and ResNet50 under two training scenarios: Kaggle‐only training with 2536 images (1268 per class) and a manually prepared combined training set with 2580 images (1290 per class), formed by adding 22 local images per class to the Kaggle training folders. Local images were linked to anonymous child identifiers before partitioning.

**Results:**

The checklist model achieved 92.5% accuracy (248/268; 95% CI, 89.6%–95.5%) and ROC‐AUC 0.984 (95% CI, 0.971–0.993) in internal out‐of‐fold evaluation. In the child‐level reanalysis, the Kaggle‐only scenario produced zero‐shot/adapted accuracies of 42.1%/68.4% for EfficientNet‐B2% and 68.4%/89.5% for ResNet50 in 19 locked test children. With the 2580‐image combined training set, zero‐shot/adapted accuracies were 71.4%/78.6% for EfficientNet‐B2% and 57.1%/85.7% for ResNet50 in 14 locked test children. These image results are preliminary and based on small local test cohorts.

**Conclusion:**

The checklist analysis showed high internal agreement with an existing psychiatrist‐assigned label, whereas image performance varied by architecture, training scenario, population transfer, and local adaptation. The results do not establish clinical validity or autonomous diagnosis. Future prospective, multicenter, participant‐level evaluation with independent reference standards is required before any clinical use.

## 1 Introduction

Autism spectrum disorder (ASD) is a heterogeneous neurodevelopmental condition characterized by persistent differences in social communication and interaction, together with restricted or repetitive patterns of behavior, interests, or activities. Its clinical presentation varies across individuals, and associated developmental, behavioral, and medical conditions may co‐occur [1]. Diagnosis is based on a comprehensive clinical assessment integrating developmental history, caregiver information, direct observation, and professional judgment; no single image, checklist, or algorithm can independently establish an ASD diagnosis. Screening instruments may identify children who warrant further assessment, but they do not establish the diagnosis by themselves. Established diagnostic criteria, including DSM‐5 criteria, guide clinical assessment rather than function as an autonomous yes/no screening tool [2]. Computer‐vision studies have explored facial images as a potentially accessible input for ASD‐related research and preliminary screening [3]. However, performance obtained from a single public image collection may not generalize to a different population or acquisition setting. Differences in demographic composition, image‐collection conditions, dataset construction, and reference‐label definition can affect the apparent performance of an image‐based model [4].

The present work evaluated two image‐training scenarios and a separate structured checklist model. EfficientNet‐B2 and ResNet50 were evaluated under Kaggle‐only training and training on a manually prepared combined dataset containing Kaggle images plus 22 local images per class. The checklist component used seven binary DSM‐5‐derived variables to model agreement with an existing psychiatrist‐assigned label. The objectives were to [1]: compare two image‐training scenarios using EfficientNet‐B2 and ResNet50 [2]; evaluate transfer from public images to local children [3]; assess whether local adaptation under child‐level partitioning improves preliminary discrimination; and [4] evaluate agreement between structured DSM‐5 checklist variables and an existing clinical label. We did not hypothesize or test that the image models could independently diagnose ASD.

## 2 Methods

### 2.1 Study Design, Setting, and Reporting Framework

This was a methodological study comprising requirements identification, retrospective development of two analytical AI components, and preliminary internal evaluation. The study was conducted within the ABBILAR [5] research program at Tabriz University of Medical Sciences. Relevant TRIPOD + AI reporting recommendations were considered when revising the description of model development, data partitioning, performance measures, uncertainty, and intended use [6].

The intended use of the present work is a research‐only methodological evaluation. The analyses are not intended to constitute a clinical decision‐support system, an autonomous diagnostic tool, or a deployable screening instrument. Any future screening‐related use would require clinician supervision, independent external validation, and prospective assessment of clinical utility.

### 2.2 Requirements Identification

A structured literature review was undertaken to identify image‐quality, technical, and clinical requirements for the study framework. The original search retrieved 979 records and included 23 publications after screening. The review informed the recording protocol, candidate image‐quality rules, model‐selection rationale, and the use of structured DSM‐5 checklist variables. The original requirements review searched PubMed, Scopus, Web of Science, SID, IranDoc, Google Scholar, and manual sources using combinations of terms for ASD, artificial intelligence, facial expression, face image, and diagnosis. The retained study record did not preserve database‐specific search dates, full syntax, or duplicate‐screening details; this is a limitation of the requirements‐identification component, which is therefore not presented as a systematic review with reproducible search methods [7].

### 2.3 Data Sources and Participants

#### 2.3.1 DSM‐5 Checklist Dataset

The structured clinical dataset contained 268 retrospective records, with 134 psychiatrist‐labeled ASD and 134 psychiatrist‐labeled non‐ASD records. Seven binary variables corresponded to the two DSM‐5 symptom domains. Each variable was coded 1 when the criterion was documented as present and 0 when it was documented as absent (Table 1). The outcome was the final psychiatrist‐assigned clinical label recorded in the clinical file (yes/no in the analytic workbook).

**Table 1:** Structured DSM‐5 checklist variables used in the logistic‐regression component.

Variable	DSM‐5 domain represented	Coding	Analytic role
A1	Deficits in social‐emotional reciprocity	1 = criterion documented present; 0 = absent	Predictor
A2	Deficits in nonverbal communicative behaviors used for social interaction	1 = present; 0 = absent	Predictor
A3	Deficits in developing, maintaining, and understanding relationships	1 = present; 0 = absent	Predictor
B1	Stereotyped or repetitive motor movements, use of objects, or speech	1 = present; 0 = absent	Predictor
B2	Insistence on sameness, inflexible adherence to routines, or ritualized patterns	1 = present; 0 = absent	Predictor
B3	Highly restricted, fixated interests abnormal in intensity or focus	1 = present; 0 = absent	Predictor
B4	Hyper‐ or hyporeactivity to sensory input or unusual sensory interests	1 = present; 0 = absent	Predictor
Psychiatrist‐assigned final label	Final clinical record label	“yes” = ASD; “no” = non‐ASD	Reference outcome

The checklist model is therefore a model of agreement with an existing clinical label based on related checklist information. It is not an independent diagnostic validation against a reference standard that is independent of the predictors. The clinical label may additionally reflect observation, interview, caregiver report, and clinical judgment not represented by the seven variables.

#### 2.3.2 Facial‐Image Dataset

The local facial‐image archive contained 162 analytic images from children aged 2–6 years: 81 ASD‐labeled and 81 non‐ASD‐labeled images. The images were linked, using a de‐identified reconstruction from the original videos and study records, to 92 unique children: 43 ASD and 49 non‐ASD. Twenty‐six children contributed one image, 62 contributed two images, and four contributed three images; the median number of images per child was two (range, 1–3). The children had a mean age of 3.85 ± 1.00 years overall, with a median age of 4 years. The ASD group had a mean age of 3.77 ± 1.04 years and included 35 boys (81.4%) and 8 girls (18.6%), whereas the non‐ASD group had a mean age of 3.92 ± 0.98 years and included 27 boys (55.1%) and 22 girls (44.9%).

The local recording protocol was developed through two focus‐group sessions and structured interviews with eight specialists, including child and adolescent psychiatry, clinical psychology, occupational therapy, neurology, speech therapy, and AI expertise. Recordings were made with an iPhone 13 at 1080p resolution and 60 frames per second. After obtaining parental consent and scheduling a time when the child was not tired, hungry, or ill, at least 3 min of child–caregiver play was recorded. The caregiver used age‐appropriate preferred toys (e.g., dolls, toy phones, toy cars, drawing materials, and blocks) to support spontaneous interaction without coercive prompting. Recording took place in an autism clinic or, for non‐ASD children, a kindergarten setting after parental and institutional permission. The room was checked for adequate light, minimal environmental noise, comfortable temperature, and limited distracting stimuli. The camera was placed at approximately 1 m, fixed at the child/caregiver eye level; flash, abrupt lighting changes, loud sounds, and sudden camera movements were avoided. If the child became distressed or unsettled, the recording was paused or rescheduled. Videos were reviewed frame by frame, and static images were eligible when the face was visible with adequate sharpness, usable frontal or near‐frontal angle, acceptable illumination, and minimal occlusion. Thirty additional files present in the local raw‐image folder were not part of the 162‐image analytic mapping and were excluded before splitting; an audit list was retained. No images from the same child were assigned to different partitions.

Public face images were obtained from the Kaggle autism collection [8]. The public training set contained 2536 images (1268 autistic and 1268 non‐autistic images), and the public validation set contained 100 images. Participant identifiers were unavailable for the public collection. Public results are therefore reported as image‐level performance within the available public evaluation protocol and are not interpreted as participant‐level clinical validation.

### 2.4 Ethical Approval, Consent, Privacy, and Data Security

The study was approved by the Student Ethics Committee of Tabriz University of Medical Sciences (IR.TBZMED.REC.1404.012). Written informed consent for participation and publication of photographs was obtained from parents or legal guardians. The analytic mapping used anonymous child research codes. The key linking research codes to identifiable source records remained at the study site and was not used in model training or transmitted with analysis outputs. Source videos were deleted from the recording phone after transfer and were stored in a dedicated folder on a personal laptop protected by a strong password. Access to the source videos was limited to the psychiatry specialist responsible for confirming image suitability. The files will be retained only until manuscript publication. This restricted‐access practice is reported transparently; however, because the files were stored on a personal device and encryption, institutional backup, and a formal audit trail were not documented, residual data‐security risks remain. Future data collection should use an institutionally managed encrypted storage system, role‐based access control, an audit trail, and a prespecified retention/deletion schedule.

Because facial images are potentially identifying biometric data, the local images, source videos, consent forms, and direct identifiers are not publicly shared. De‐identified code, model configuration, split‐assignment logic, and non‐identifying prediction outputs can be shared on reasonable request subject to ethics approval and data‐governance requirements.

### 2.5 Image Preprocessing and Child‐Level Partitioning

Image filenames were linked to anonymous child identifiers before splitting. A seeded, stratified partition at the child level was created using random seed 42: 55 children (95 images; 26 ASD and 29 non‐ASD) were assigned to local adaptation training, 18 children (33 images; 8 ASD and 10 non‐ASD) to validation, and 19 children (34 images; 9 ASD and 10 non‐ASD) to locked testing. The locked‐test children were not used for local adaptation, validation‐based checkpoint selection, threshold selection, or model selection.

For local adaptation, training images were resized to 256 × 256 pixels and augmented using random resized crops (scale, 0.85–1.00), horizontal flips (probability, 0.30), brightness/contrast jitter, and rotation (±8°). Evaluation images were resized to 256 × 256 pixels and center‐cropped without stochastic augmentation. All images were normalized using ImageNet channel means (0.485, 0.456, 0.406) and standard deviations (0.229, 0.224, 0.225).

### 2.6 Image Model Development, Two Training Scenarios, and Local Adaptation

Two image‐training scenarios were defined to examine the effect of incorporating local facial images into the public training data. The same experimental logic was applied separately to EfficientNet‐B2 and ResNet50, resulting in four model–scenario configurations. In Scenario 1 (Kaggle‐only training), each model was trained exclusively on the public Kaggle autism image collection. The training set contained 2536 images, comprising 1268 ASD and 1268 non‐ASD images; the public validation set contained 100 images. The local archive contained 162 images from 92 children and was not used to train the initial Kaggle‐only checkpoint. In Scenario 2 (combined Kaggle–local training), 22 ASD and 22 non‐ASD local images were manually added to the corresponding Kaggle training folders before model training. This produced a combined training set of 2580 images, with 1290 images per class. The 44 transferred local images were excluded from local evaluation, leaving 118 local images (59 ASD and 59 non‐ASD) for the combined‐scenario evaluation. The children contributing to the local training and local evaluation subsets were non‐overlapping according to their anonymous child identifiers.

For both scenarios, EfficientNet‐B2 and ResNet50 were initialized with ImageNet‐1K weights. Each model produced two output logits corresponding to the ASD and non‐ASD classes. The classification heads and the layers updated during local adaptation were specified separately for each architecture in the implementation.

For public and combined‐dataset training, the optimizer was AdamW with an initial learning rate of 2 × 10^−4^ and a weight decay of 1 × 10^−5^. The main training loop used a batch size of 8 and a maximum of 40 epochs. Cross‐entropy loss with label smoothing of 0.10 was used. MixUp augmentation was applied with a probability of 0.70 using a Beta distribution parameter of α = 0.20. Gradient norms were clipped at 1.0, and the learning rate was updated using a cosine‐annealing schedule with a maximum period corresponding to the 40 training epochs. The checkpoint with the highest validation accuracy was retained separately for each architecture and training scenario.

Early stopping in the main training loop was based on validation accuracy rather than validation loss. Training was terminated when the validation accuracy failed to improve for 12 consecutive epochs. Thus, the patience value was 12 epochs, and the model state with the highest validation accuracy was restored for final evaluation.

During local fine‐tuning, the local data were divided into training and validation subsets, and both the training and validation data loaders used a batch size of 4. Fine‐tuning was performed for up to 15 epochs using AdamW with a learning rate of 1 × 10^−4^ and a weight decay of 1 × 10^−5^. A class‐weighted two‐class cross‐entropy loss was used to account for the class distribution in the local training subset, together with a cosine‐annealing learning‐rate schedule. The trainable parameters consisted of the classification head and, where applicable, the designated final feature layers. The model state with the highest local validation accuracy was restored before subsequent evaluation. No separate early‐stopping rule based on local validation loss was implemented during this fine‐tuning procedure.

A subsequent child‐level reanalysis was conducted for both architectures and both training scenarios to evaluate public‐to‐local transfer and local adaptation while avoiding image‐level leakage. Before partitioning, every local image was linked to an anonymous child identifier. All images belonging to the same child were assigned to only one partition. In the Kaggle‐only analysis, the 162‐image local archive was divided into child‐level training, validation, and locked‐test partitions comprising 55, 18, and 19 children, respectively. In the combined‐training analysis, the 118 local images remaining after the removal of the 44 transferred training images were divided into child‐level training, validation, and locked‐test partitions comprising 42, 14, and 14 children, respectively.

For the zero‐shot evaluation, the public or combined‐dataset checkpoint was applied to the locked local test children without local fine‐tuning. For the local‐adaptation evaluation, the designated trainable feature layers and classification head were updated using the local training children. The best checkpoint was selected using local validation accuracy, and the probability threshold was selected using the validation children only. The locked‐test children were not used for local adaptation, checkpoint selection, threshold selection, or model selection and were evaluated only once at the end of the analysis. Child‐level ASD probabilities were calculated by averaging the image‐level ASD probabilities for each child.

Hyperparameters were selected manually during feasibility development from commonly used transfer‐learning values and then fixed before the reanalysis. No grid search, random search, Bayesian optimization, or test‐set‐driven tuning was performed. Accordingly, the comparison between EfficientNet‐B2 and ResNet50 and between the two training scenarios is exploratory and preliminary rather than an optimized or definitive model comparison.

### 2.7 DSM‐5 Checklist Model

Logistic regression was selected because it provides a transparent baseline for structured binary checklist variables. The model used the seven features in Table 1, L2 regularization with the liblinear solver, maximum 2000 iterations, random seed 42, and a probability threshold of 0.50. Performance was assessed from stratified 10‐fold out‐of‐fold predictions across all 268 records. A secondary 70:30 stratified split was conducted as a sensitivity analysis, not as the primary result.

### 2.8 Statistical Analysis

All analyses were performed in Python 3.12.13 using PyTorch 2.10.0 + cu128, torchvision 0.25.0 + cu128, NumPy 2.0.2, pandas 2.3.3, and scikit‐learn 1.6.1 on a Kaggle Tesla T4 GPU. For the earlier DSM‐5 reanalysis, Python 3.12.3, pandas 3.0.5, NumPy 2.5.1, and scikit‐learn 1.9.0 were used; the final numerical results are reported with the specific analysis output.

For a binary outcome, let TP, TN, FP, and FN denote true positives, true negatives, false positives, and false negatives, respectively. We calculated:

Accuracy = (TP + TN)/(TP + TN + FP + FN).

Sensitivity = TP/(TP + FN); Specificity = TN/(TN + FP).

Precision (positive predictive value) = TP/(TP + FP); F1 = 2 × Precision × Sensitivity/(Precision + Sensitivity).

A ROC curve plots sensitivity against 1−specificity across all possible thresholds; its area (ROC‐AUC) summarizes discrimination. A precision–recall curve plots precision against sensitivity, and average precision summarizes this curve. The Brier score was calculated as:

Brier score = (1/*n*) × Σ*ᵢ*_ = 1_^*n*^ (*pᵢ* − *yᵢ*)^2^.

where *pᵢ* is the predicted ASD probability and *yᵢ* ∈ {0,1} is the observed ASD label for individual *i*. For the image analysis, n denotes the number of evaluated children rather than the number of images. At the child level, *pᵢ* was the arithmetic mean of all image‐level ASD probabilities for that child.

For the local locked test set, 95% CIs were percentile intervals from 2000 stratified nonparametric bootstrap resamples of children within outcome class. The DSM‐5 CIs used 2000 stratified bootstrap resamples of the out‐of‐fold predictions. No *p* values or formal between‐model hypothesis tests were reported because the locked local test sets included only 19 and 14 children, respectively, and the analysis was designed as a preliminary post hoc leakage audit rather than a prespecified comparative trial. ROC and precision–recall curves were generated for both local scenarios; calibration plots are descriptive because of the small test samples.

The overall execution process of the present study is illustrated in Figure 1.

**Figure 1:**
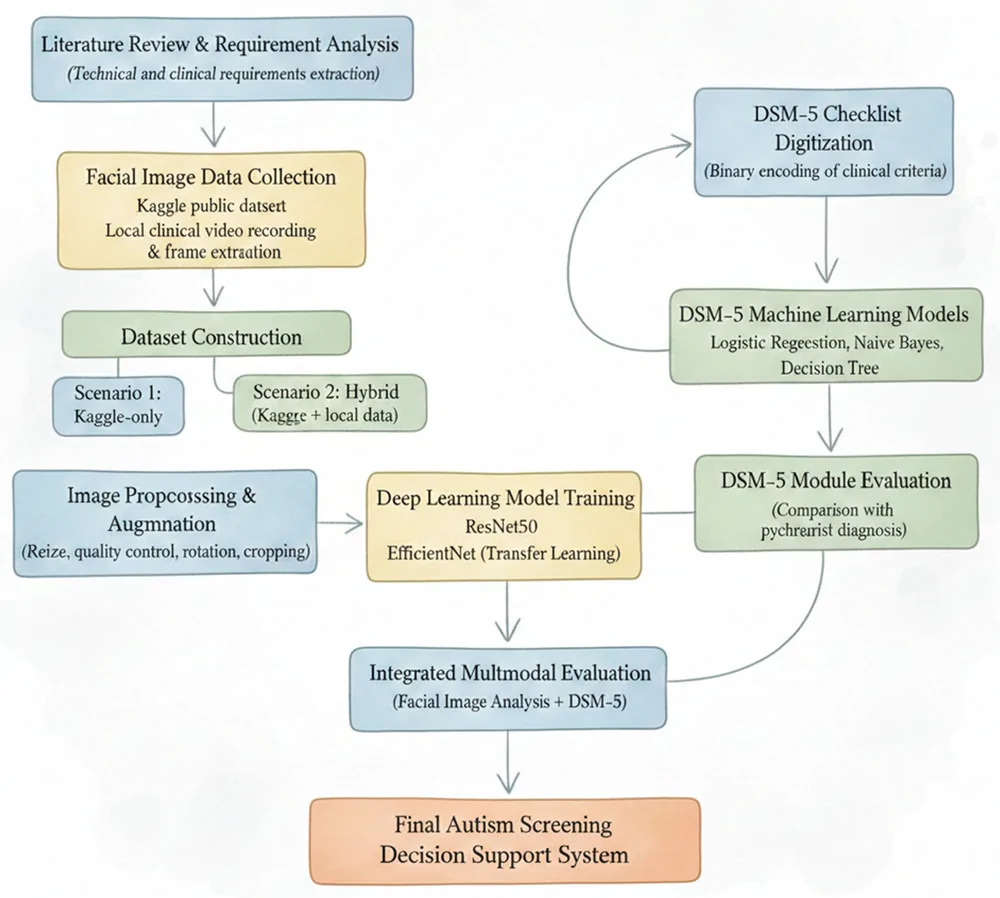
Flowchart of the complete methodological pipeline of the study.

## 3 Results

### 3.1 Requirements Identification

The requirement‐identification review screened 979 records and retained 23 publications. Across the reviewed studies, common requirements included frontal or near‐frontal facial visibility, adequate illumination, limited occlusion, appropriate image resolution, explicit preprocessing, transparent separation of development and evaluation samples, and caution when transferring models across image‐acquisition settings. The review informed the local recording protocol and the present reanalysis.

### 3.2 Checklist Dataset and DSM‐5 Model

The DSM‐5 dataset included 268 records with equal ASD and non‐ASD class counts (134/268 [50.0%] in each group). No value was missing for any of the seven binary variables. The logistic‐regression model obtained 248/268 correct out‐of‐fold classifications (92.5%; 95% CI, 89.6%–95.5%). The confusion matrix was TN = 119, FP = 15, FN = 5, and TP = 129. Sensitivity was 96.3% (129/134), specificity was 88.8% (119/134), precision was 89.6% (129/144), F1 score was 92.8%, ROC‐AUC was 0.984 (95% CI, 0.971–0.993), average precision was 0.985, and the Brier score was 0.066. The 70:30 sensitivity analysis yielded 75/81 correct classifications (92.6%; 95% CI, 86.4%–97.5%) and ROC‐AUC 0.990.

These results quantify agreement with the psychiatrist‐assigned reference label; they do not provide independent diagnostic accuracy because the checklist features and clinical label originate from related clinical assessment processes.

Figure 2 illustrates the out‐of‐fold evaluation of the logistic‐regression model based on the structured DSM‐5 checklist variables.

**Figure 2:**
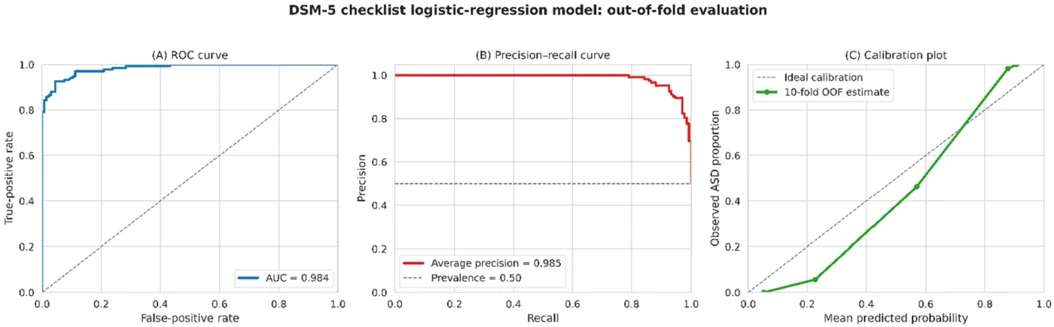
DSM‐5 checklist logistic‐regression out‐of‐fold evaluation.

### 3.3 Child‐Level Facial‐Image Analysis

Table 2 summarizes the child‐level partition.

**Table 2:** Local facial‐image cohort and participant‐level data partition.

Analysis/scenario	ASD children	non‐ASD children	Total children	ASD images	non‐ASD images	Total images
Kaggle‐only child split: train	26	29	55	49	46	95
Kaggle‐only child split: validation	8	10	18	15	18	33
Kaggle‐only child split: locked test	9	10	19	17	17	34
Combined‐training child split: train	19	23	42	36	37	73
Combined‐training child split: validation	7	7	14	12	11	23
Combined‐training child split: locked test	6	8	14	11	11	22
Kaggle‐only local pool: total	43	49	92	81	81	162
Combined‐training local pool: total	32	38	70	59	59	118

The table describes only the local data used for child‐level reanalysis; it does not represent the size of the Kaggle or combined training folders. In Scenario 1, the complete local archive contained 162 images. In Scenario 2, 44 local images were incorporated into the combined training set and the remaining 118 local images were used for local evaluation. Each child contributed to only one partition.

The local facial‐image archive initially contained 162 images, including 81 ASD‐labeled and 81 non‐ASD‐labeled images. In Scenario 1, all 162 images formed the local evaluation pool before child‐level partitioning. In Scenario 2, 22 ASD and 22 non‐ASD images were incorporated into the 2580‐image combined training folders, leaving 118 local images for evaluation. These local evaluation images were partitioned by anonymous child identifier into training, validation, and locked‐test subsets. The 19‐child and 14‐child counts reported below refer only to the locked‐test subsets used in the reanalysis.

To address the possibility of image‐level dependence, a separate child‐level reanalysis was performed for each training scenario and architecture. In Scenario 1, the 162‐image local evaluation pool was partitioned by child identifier into a local‐adaptation training subset, a validation subset, and a locked test subset. The locked test subset contained 19 children and 34 images, including 9 ASD children contributing 17 images and 10 non‐ASD children contributing 17 images. In Scenario 2, the remaining 118 local evaluation images were partitioned using the same child‐level procedure. The resulting locked test subset contained 14 children and 22 images, including 6 ASD children contributing 11 images and 8 non‐ASD children contributing 11 images. In both scenarios, images from the same child were assigned to only one partition. The locked test children were not used for local adaptation, early stopping, threshold selection, or model selection.

All image‐level ASD probabilities were aggregated at the child level before the final performance metrics were calculated. For children contributing more than one image, the arithmetic mean of the image‐level ASD probabilities was used as the child‐level probability. Accordingly, accuracy, sensitivity, specificity, F1 score, ROC‐AUC, average precision, and Brier score were calculated from one final prediction per child rather than from individual images.

### 3.4 EfficientNet‐B2

In Scenario 1, the Kaggle‐only EfficientNet‐B2 model correctly classified 8 of 19 locked test children in the zero‐shot evaluation, corresponding to an accuracy of 42.1% and a ROC‐AUC of 0.356. After local adaptation using only the local training children, the model correctly classified 13 of 19 locked test children, corresponding to an accuracy of 68.4% and a ROC‐AUC of 0.778. The adapted analysis produced a sensitivity of 66.7%, a specificity of 70.0%, an F1 score of 66.7%, an average precision of 0.816, and a Brier score of 0.189. The corresponding zero‐shot values were 44.4% sensitivity, 40.0% specificity, an F1 score of 42.1%, an average precision of 0.416, and a Brier score of 0.500.

In Scenario 2, which used the 2580‐image combined training set, EfficientNet‐B2 correctly classified 10 of 14 locked test children without local adaptation, corresponding to an accuracy of 71.4% and a ROC‐AUC of 0.750. Following local adaptation with the local training children, 11 of 14 locked test children were correctly classified, corresponding to an accuracy of 78.6% and a ROC‐AUC of 0.938. The adapted analysis yielded a sensitivity of 66.7%, a specificity of 87.5%, an F1 score of 72.7%, an average precision of 0.931, and a Brier score of 0.114.

### 3.5 ResNet50

In Scenario 1, the Kaggle‐only ResNet50 model correctly classified 13 of 19 locked test children in the zero‐shot evaluation, corresponding to an accuracy of 68.4% and a ROC‐AUC of 0.689. After local adaptation, it correctly classified 17 of 19 locked test children, corresponding to an accuracy of 89.5% and a ROC‐AUC of 0.922. The adapted analysis yielded a sensitivity of 88.9%, a specificity of 90.0%, an F1 score of 88.9%, an average precision of 0.943, and a Brier score of 0.107. The corresponding zero‐shot values were 66.7% sensitivity, 70.0% specificity, an F1 score of 66.7%, an average precision of 0.733, and a Brier score of 0.457 (Table 3).

**Table 3:** Child‐level locked‐test performance of the facial‐image component.

Model/scenario/status	Correct/total	Accuracy %	Sensitivity %	Specificity %	Precision %	F1%	ROC‐AUC	Average precision	Brier score
EfficientNet‐B2; Kaggle‐only; zero‐shot	8/19	42.1	44.4	40.0	40.0	42.1	0.356	0.416	0.500
EfficientNet‐B2; Kaggle‐only; adapted	13/19	68.4	66.7	70.0	66.7	66.7	0.778	0.816	0.189
ResNet50; Kaggle‐only; zero‐shot	13/19	68.4	66.7	70.0	66.7	66.7	0.689	0.733	0.457
ResNet50; Kaggle‐only; adapted	17/19	89.5	88.9	90.0	88.9	88.9	0.922	0.943	0.107
EfficientNet‐B2; combined; zero‐shot	10/14	71.4	50.0	87.5	75.0	60.0	0.750	0.702	0.272
EfficientNet‐B2; combined; adapted	11/14	78.6	66.7	87.5	80.0	72.7	0.938	0.931	0.114
ResNet50; combined; zero‐shot	8/14	57.1	83.3	37.5	50.0	62.5	0.563	0.494	0.292
ResNet50; combined; adapted	12/14	85.7	83.3	87.5	83.3	83.3	0.958	0.948	0.087

In Scenario 2, ResNet50 correctly classified 8 of 14 locked test children without local adaptation, corresponding to an accuracy of 57.1% and a ROC‐AUC of 0.563. After local adaptation using only the local training children, it correctly classified 12 of 14 locked test children, corresponding to an accuracy of 85.7% and a ROC‐AUC of 0.958. The adapted analysis yielded a sensitivity of 83.3%, a specificity of 87.5%, an F1 score of 83.3%, an average precision of 0.948, and a Brier score of 0.087.

Table 3 shows the child‐level locked‐test evaluation for EfficientNet‐B2 and ResNet50 under the Kaggle‐only and combined‐training scenarios. ROC, precision–recall, and calibration curves are shown separately for zero‐shot and locally adapted predictions. Thresholds were selected using validation children only; locked‐test children were not used for adaptation, early stopping, threshold selection, or model selection.

The ROC, precision–recall, and descriptive calibration curves for the locally adapted EfficientNet‐B2 model under the Kaggle‐only scenario are shown in Figure 3.

**Figure 3:**
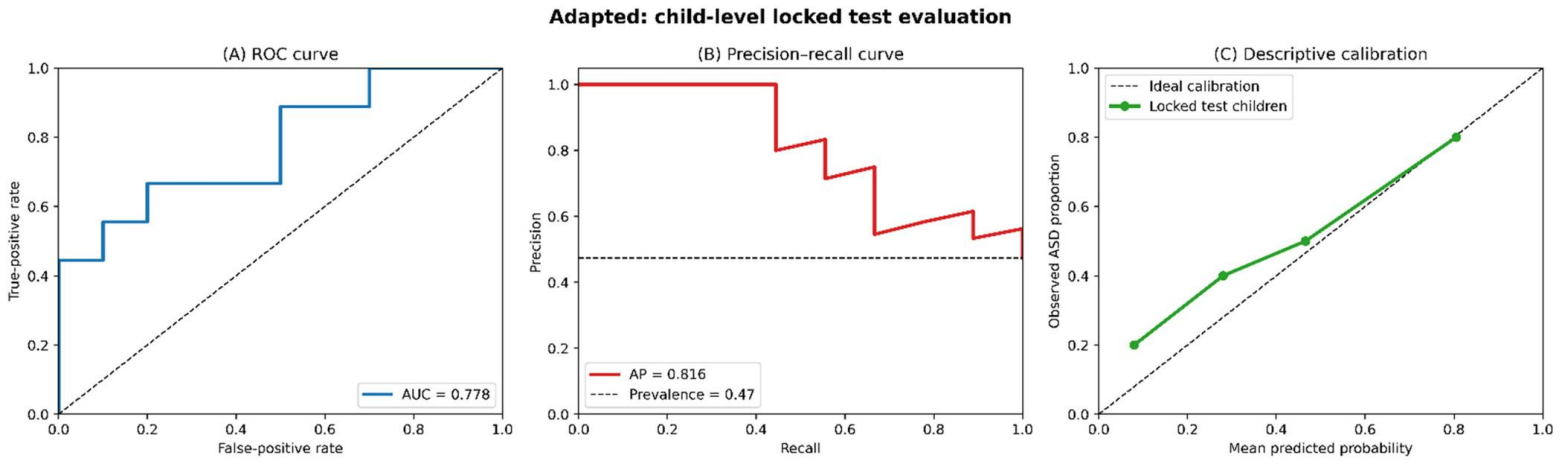
Child‐level locked‐test evaluation of the adapted EfficientNet‐B2 model in Scenario 1 (Kaggle‐only training). The model was adapted using only local training children, and the decision threshold was selected using validation children. Panels A–C show the ROC curve (ROC‐AUC = 0.778), precision–recall curve (average precision = 0.816), and descriptive calibration plot, respectively.

The 19‐child and 14‐child counts refer only to the locked‐test subsets used in the child‐level reanalysis; they do not represent the total number of local images or the full local archive. Scenario 1 used a 162‐image local evaluation pool, whereas Scenario 2 used the 118 local images remaining after 44 local images were incorporated into the combined training set. Because the scenarios used different local evaluation pools, their child‐level performance estimates should not be interpreted as a direct comparison on identical test cohorts. The results are exploratory and should not be interpreted as evidence of clinical effectiveness, diagnostic validity, or superiority of one architecture. The small number of locked‐test children resulted in wide uncertainty intervals, and no formal hypothesis tests were conducted between models or training scenarios.

Table 4 places the present study alongside representative facial‐image, hybrid, eye‐tracking, environmental, and other AI‐based ASD studies. Direct numerical comparison is limited by differences in population, class definitions, image source, sample size, reference standard, participant‐level separation, model architecture, and evaluation protocol. In particular, high image‐level accuracy obtained from public or curated datasets should not be interpreted as evidence of clinical generalizability. The present analysis illustrates why the composition of the training data, the distinction between image‐level and child‐level evaluation, local adaptation, and independent participant‐level testing should be reported explicitly.

**Table 4:** Contextual comparison with selected ASD AI studies.

Study	Modality	Design/evaluation context	Main method	Interpretation for present study
Awaji et al. [3]	Facial images	Curated image dataset	CNN feature combination	Demonstrates hybrid deep‐feature approaches; participant‐level separation must be checked before comparing accuracy.
Sariyanidi et al. [9]	Facial behavior	Human–AI comparison	AI prediction from facial behavior	Highlights clinical‐context and human‐comparator issues.
Balasubramani, Surendran [10]	Multimodal signals	ASD classification	Explainable multimodal quantum learning	Conceptually relevant to future data fusion; not directly comparable with facial still images.
Present study	Facial images and separate DSM‐5 checklist component	Retrospective methodological study; child‐level local reanalysis	EfficientNet‐B2, ResNet50; logistic regression	Reports public‐to‐local transfer, training‐data composition, local adaptation, and checklist‐label agreement rather than autonomous diagnosis.

## 4 Discussion

This study evaluated two image‐training scenarios with two CNN architectures and a separate structured checklist model. The central finding is that image performance depended strongly on training‐data composition, architecture, and evaluation level. The child‐level reanalysis showed poor‐to‐moderate transfer without adaptation and variable improvement after adaptation, while the checklist model showed high agreement with an existing clinical label.

The DSM‐5 logistic‐regression result should be interpreted as agreement with a related reference label, not as independent validation of psychiatric diagnosis. The image results likewise should not be interpreted as an autonomous ASD diagnosis. The two image‐training scenarios were not fused with checklist data because paired participant‐level image–checklist data were unavailable.

The child‐level reanalysis altered the interpretation of the original exploratory findings by preventing images from the same child from appearing in different partitions. Under Kaggle‐only training, EfficientNet‐B2 improved from 42.1% zero‐shot accuracy to 68.4% after adaptation, while ResNet50 improved from 68.4% to 89.5%. Under combined training, EfficientNet‐B2 changed from 71.4% to 78.6%, and ResNet50 from 57.1% to 85.7%. These estimates are preliminary because the locked test sets contained only 19 and 14 children, respectively.

The primary purpose of the present image analysis was not to determine whether facial images are superior to other ASD‐related modalities. Instead, it was to examine how training‐data composition and local adaptation affected the transfer of image‐based models from a public dataset to an independent local child cohort. Previous facial‐image studies have often focused on within‐dataset discrimination, whereas the present analysis additionally examined public‐to‐local transfer and participant‐level separation [7]. A model may achieve high accuracy on a curated image dataset while showing reduced performance on an independent local cohort because of differences in population, image quality, background, lighting, camera conditions, or participant characteristics. Accordingly, direct numerical comparison with other studies is inappropriate unless the population, reference standard, dataset construction, participant‐level separation, and evaluation protocol are sufficiently comparable.

Differences in ethnicity, lighting, background, facial pose, and image quality may lead a model to learn dataset‐specific patterns rather than clinically relevant facial characteristics. The improvement after local adaptation supports the findings of recent domain‐adaptation studies, which have shown that incorporating target‐population data can improve robustness across datasets [11].

Studies using other modalities address related but different scientific questions. For example, a multimodal approach combines information from several data sources and therefore evaluates whether heterogeneous signals can be integrated to improve prediction [10].

Direct numerical comparison with studies based on other data types or clinical objectives is therefore inappropriate. Differences in population, image‐acquisition conditions, sample size, class definition, reference labels, participant‐level separation, and evaluation protocol can substantially affect reported performance. In particular, high accuracy obtained from a curated public dataset does not necessarily indicate that a model will perform reliably on images collected from an independent local population. The contrast between the public‐data results and the local child‐level results in the present study illustrates this distinction. Accordingly, the contribution of this work is methodological rather than a definitive clinical performance claim: it highlights the importance of examining training‐data composition, public‐to‐local transfer, participant‐level separation, and local adaptation before the broader applicability of facial‐image models is considered.

The DSM‐5 logistic‐regression model correctly classified 248 of 268 clinical records, achieving 92.5% accuracy, 96.3% sensitivity, 88.8% specificity, and a ROC‐AUC of 0.984. This result indicates agreement between the structured checklist variables and the psychiatrist‐assigned clinical label. It does not provide independent validation of the DSM‐5 criteria or a replacement for psychiatric assessment because the checklist variables and clinical labels originated from related clinical records. The checklist and facial‐image analyses should therefore be regarded as separate research components rather than as equivalent diagnostic tests or a fused multimodal system.

Explainability remains an important requirement for the clinical translation of image‐based models. Grad‐CAM, SHAP, LIME, or related techniques were not prespecified or completed in the present reanalysis. Accordingly, this study does not claim that any particular facial region contributed clinically meaningful information to the model predictions. Future investigations should define explainability procedures before model evaluation and should assess whether attribution maps focus on plausible facial regions rather than background features, image artifacts, or acquisition‐related characteristics.

The study has several limitations. First, the local image cohorts and locked test partitions were small, producing wide confidence intervals. Second, the child‐level reanalysis was conducted after the original exploratory workflow and should be considered a robustness audit. Third, public participant identifiers were unavailable. Fourth, the combined training set was prepared manually and the provenance of individual transferred images should be documented in a reproducible data manifest. Fifth, no independent external cohort or prospective clinical‐impact study was available. Finally, image‐based assessment raises privacy, consent, stigmatization, and misuse concerns; the models must not be used for autonomous diagnosis or exclusionary decisions.

## 5 Conclusion and Future Work

This study conducted a methodological evaluation of two image‐training scenarios using EfficientNet‐B2 and ResNet50, together with a separate analysis of structured DSM‐5 checklist variables. The image analyses compared models trained on the public Kaggle dataset with models trained after the addition of local images, and further examined public‐to‐local transfer and local adaptation under participant‐level separation. Performance differed across architectures and training‐data configurations, indicating that results obtained from a public image dataset may not be preserved when models are applied to an independent local cohort. The child‐level locked‐test analyses should therefore be interpreted as preliminary estimates of transportability rather than as evidence of diagnostic accuracy or clinical effectiveness.

The checklist analysis showed substantial agreement with the existing psychiatrist‐assigned clinical label; however, this model should be interpreted as a model of agreement with an available clinical label rather than as an independent diagnostic system.

Future work should prospectively recruit a larger, multicenter cohort with participant‐level identifiers; prespecify model selection and evaluation; retain age, sex, and acquisition metadata; document the provenance of manually combined training data; assess subgroup fairness; use independently adjudicated reference standards; quantify calibration and clinical utility; compare CNN and transformer architectures under the same partition; conduct clinician‐reviewed explainability analyses; and assess whether any tool improves referral timeliness without increasing false‐positive burden. Until then, the models should remain research‐only analytical methods.

## Author Contributions

**Fatemeh Mohammadi:** conceptualization, methodology, software, data curation, investigation, formal analysis, visualization, writing – original draft, writing – review and editing. **Peyman Rezaei‐Hachesu:** conceptualization, methodology, investigation, validation, formal analysis, visualization, project administration, writing – review and editing. **Afsoon Asadzadeh:** conceptualization, methodology, investigation, formal analysis, supervision, resources, visualization, writing – review and editing. **Hassan Shahrokhi:** conceptualization, methodology, data curation, investigation, validation, formal analysis, supervision, visualization, project administration, writing – review and editing. All authors approved the final version of the manuscript and agreed to be accountable for all aspects of the work.

## Funding

This research received no specific grant from any funding agency in the public, commercial, or not‐for‐profit sectors. No external funder had any role in study design; data collection, analysis, or interpretation; manuscript writing; or the decision to submit the manuscript for publication.

## Ethics Statement

The study was approved by the Student Ethics Committee of Tabriz University of Medical Sciences (IR.TBZMED.REC.1404.012).

## Consent

Written informed consent was obtained from the parents or legal guardians of all child participants.

Parents or legal guardians provided written consent for publication of facial photographs where applicable. No identifiable facial image is included in the manuscript or Supporting files without explicit publication consent.

## Conflicts of Interest

The authors declare no conflicts of interest.

## Use of Artificial Intelligence Tools

During the preparation of this manuscript, the authors used Manus for translation/editing of an original Persian‐language draft into English. The authors reviewed, edited, and take full responsibility for all translated content. No generative AI tool was used to generate or alter research data, perform unverified statistical analysis, or create the study figures.

## Transparency Statement

All authors have read and approved the final version of the manuscript. Hassan Shahrokhi had full access to all of the data in this study and takes complete responsibility for the integrity of the data and the accuracy of the data analysis. All authors affirm that this manuscript is an honest, accurate, and transparent account of the study being reported; that no important aspects of the study have been omitted; and that any discrepancies from the study as planned have been explained.

## Data Availability

The de‐identified DSM‐5 analytic table, model configuration, and non‐identifying evaluation outputs are available from the corresponding author on reasonable request, subject to ethical approval and institutional data‐governance requirements. Local raw facial images, videos, and identifiable clinical records are not publicly available because they contain sensitive biometric and pediatric health information.
